# Understanding the influence of cation and anion migration on perovskite light-emitting diodes via transient response

**DOI:** 10.1038/s41598-023-42933-1

**Published:** 2023-09-20

**Authors:** Paria Forozi Sowmeeh, Mohammad Zohorfazeli, Elnaz Yazdani

**Affiliations:** Department of Physics, Faculty of Basic Sciences, Tarbiat Modares University, P.O. Box 14115-175, Tehran, Iran

**Keywords:** Lasers, LEDs and light sources, Photonic devices

## Abstract

Despite the rapid progress demonstrated in the efficiency of *Perovskite light*-*emitting diodes* (PeLEDs) in the past few years, ion migration has challenged the practical applications of these devices with undesirable hysteresis and degradation effect. Mobile ions in PeLEDs induced many unique and fast transient phenomena occurring on the time scale of microseconds to seconds and it is still far from clear how the underlying physical mechanism of ion motion-induced variation relates to the device performance. Therefore, in this work, we employ an ionic Drift–Diffusion Model (DDM) to evaluate measuring transient current response in a time scale of sub-seconds. The results show that spatial redistribution of ions within the perovskite results in dynamic electric field variation, which in turn, affects charge carrier injection and distribution. Moreover, the time delay between anion and cation migration leads to an unequal rate of charge carrier injection, hence the multi-stage behavior of the current–time response. It is also realized that the potential barrier of charge injection due to cation and anion accumulation at perovskite interfaces with electron and hole transporting layers reduces. Therefore, the facilitation of charge injection favors radiative recombination, and improved IQEs are expected at higher ion densities. It is found that the current–time response of the device gives beneficial information on cation and anion migration time scales. Choosing an appropriate scan rate in accordance with cation-related slow migration time is the first step to achieving reliable measurement procedures and hysteresis-free PeLED.

## Introduction

Excellent properties of metal halide perovskites such as high photoluminescence quantum efficiencies, tunable emission spectra, and solution processability make them promising light-emitting materials for low-cost and high-performance light-emitting technology. In the last few years, external quantum efficiencies of over 20% have been achieved for perovskite LEDs (PeLEDs)^1–3^. However, the intrinsic instability caused by ion migration in metal halide perovskites remains a major limiting factor in the practical applications of perovskite-based devices. Hence, the ions migration dynamics and their restricted effect on the charge carrier transportation can explain the undesirable hysteresis behavior in these devices. The J-V hysteresis in perovskite solar cells has been extensively investigated and mainly attributed to ion migration. For PeLEDs, there are only a few experimental works that have studied the ion migration effect on diodes operation^4–7^. The impact of ion migration on PeLEDs is believed to be more significant than on solar cells^1,4,6,8^ and, it has been realized that, due to the ionic nature of the perovskite materials, the characterization of the PeLEDs is often sensitive to the measurement procedures^5^. Since hysteresis is proven to be a slow process occurring at the order of a few milliseconds^9^, the ionic species with migration time scale at that range will be responsible for it. Besides, Scan rate is recognized to be a crucial parameter affecting the reported hysteresis in perovskite-based devices hence, significant introduced hysteresis is expected at scan rates matching the migration rate of the slowest ionic specie^10^. Lack of knowledge in choosing an appropriate scan rate for different perovskite compositions leads to dealing with a lot of unreliable and incomparable reported hysteresis data. We believe, revealing the migration time scale of all ionic species is the essential first step for determining an appropriate scan rate to avoid the above-mentioned problems and achieving hysteresis-free perovskite-based devices. Herein, to realize the migration time scale of each ionic species and how different ion migration time scales can affect the hysteric behavior of PeLEDs, the time transient responses of the cations and anions under applied bias using the Drift–Diffusion Model (DDM) have been studied. Due to the fast dynamics of the perovskite LEDs operation, the main simulation results have been conducted for physics occurring on time scales of a microsecond to seconds. We speculate that the time delay between electron and hole injection is the main root of the hysteresis in PeLEDs where the ionic nature of perovskites worsens the situation and makes the characterization of perovskite-based devices sensitive to the measurement procedures^5,11,12^. Ionic motion modifies the electric field and affects the charge carrier injection. Moreover, several experimental and theoretical investigations have demonstrated that hysteresis in perovskite-based devices originated from changes in the electron transport layer (ETL) and hole transport layer (HTL) interfaces and slow ionic motion^13–15^. Evidence suggests that scan parameters, particularly scan rate, have a significant impact on hysteresis^5,16^. Achieving a comprehensive picture of ion migration-induced phenomena in the sub-second regime helps to come up with an appropriate scan rate to minimize the hysteresis in PeLEDs.

## Numerical modeling

**Figure 1:**
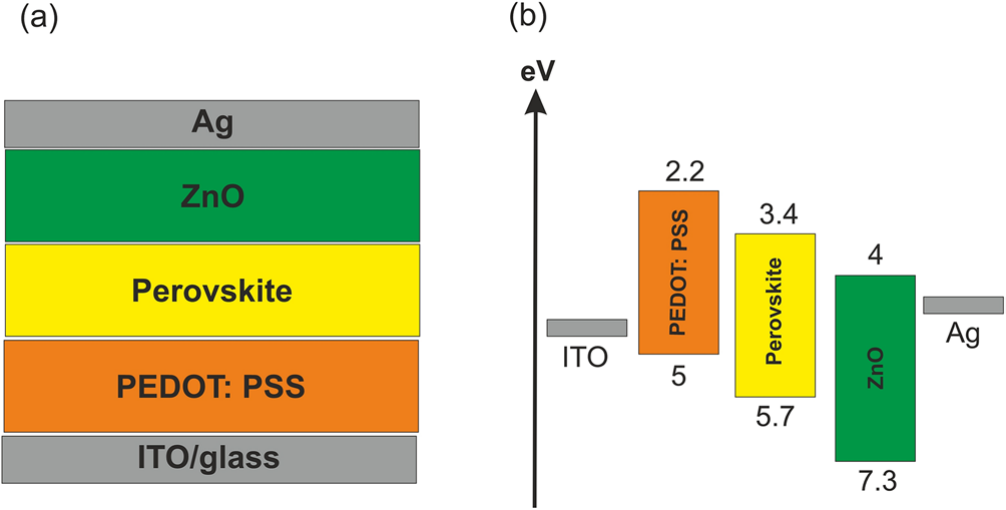
(**a**) The structure; and (**b**) The band diagram of the PeLED.

In this work, the numerical solution of the time-dependent Drift–Diffusion Model (DDM) has been used for the extraction of semiconductor device parameters^17,18^. Our model is developed to include terms for two mobile ionic species. The ionic species (“anion” and “cation" used here to refer to negative and positive ions, respectively) are included in the model as charge carriers that can migrate within the bulk material in response to the electric field and concentration gradient. They do not participate in any generation or recombination process due to their intrinsic characteristics. The ions are confined to the perovskite layer, and the transport layer interfaces completely block the ion movement. In the first place, to investigate the compatibility of our findings with those of other researchers, we compared the resultant J-t curve with references which also exhibits a similar trend with minor discrepancy resulting from applying a step voltage of the PeLED threshold^7,19^. Figure 1 represents a schematic of the PeLED layout along with the corresponding band diagram. It consists of the following structure: ITO/PEDOT:PSS/MAPbBr_3_/ZnO/Ag, where PEDOT:PSS and ZnO are HTL and ETL, respectively. The charge carrier injection and recombination in HTL, perovskite, and ETL were performed by drift–diffusion calculations. Band-to-band recombination with a carrier lifetime of 20 ns brings about the emission of green light within the perovskite. Shockley–Read–Hall (SRH) (with mid-gap defect states) and Auger recombination for charge carriers are assumed in all three layers. Moreover, interfacial defects leading to surface recombination with a velocity of 10^7^ cm/s are considered at Schottky contacts.

The following equations are the fundamental blocks in DDM:

1 $$\nabla .\left(-{\varepsilon }_{r}\nabla \mathrm{V}\right)=\mathrm{q}\left(\mathrm{p}-\mathrm{n}+{N}_{d}^{+}-{N}_{a}^{-}\right)\mathrm{ Poisson's\,Eq}.$$

2 $$\partial n/\partial t=\frac{1}{q}\left(\nabla .{J}_{n}\right)-R\,\,\,\,\,\,\,\,\mathrm{Continuity\,Eq}.\mathrm{\,for\,electrons}$$

3 $$\partial p/\partial t=-\frac{1}{q}\left(\nabla .{J}_{p}\right)-R\,\,\,\,\,\,\,\,\mathrm{Continuity\,Eq}.\mathrm{\,for\,hole}$$

where ${\varepsilon }_{r}$ is relative permittivity and $\mathrm{V}$ is electric potential. Elementary particle, density of mobile holes, electrons, donners and, acceptors are represented as $\mathrm{q},\mathrm{ p},\mathrm{n}, {N}_{d}^{+}, {N}_{a}^{-}$, respectively. ${J}_{n}$ and ${J}_{p}$ are electron and hole current density. Besides, R represents the net recombination rate.

Assuming space charge density of $q\left(c-a\right)$ within the perovskite, ion migration as a set of partial differential equations (PDE) is introduced to DDM:

4 $$\frac{\partial a}{\partial t}+\nabla .\left(-a{\mu }_{a}E-ax{D}_{a}\right)=0$$

5 $$\frac{\partial c}{\partial t}+\nabla .\left(c{\mu }_{c}E-cx{D}_{c}\right)=0$$

where $a$, $c$ and $E$ are anion and cation density and electric field. ${\mu }_{a}, {\mu }_{c},$ ${D}_{a},$ and ${D}_{c}$ are anion and cation mobility and diffusion constant. Anions and cations cannot pass through the perovskite. Hence their mobility outside of the perovskite is zero. Moreover, quasi-neutrality is considered within the perovskite layer by N_a_ = $\frac{1}{L\_perovskite}\int a\left(x\right) dx-NI$ and N_c_ = $\frac{1}{L\_perovskite}\int c\left(x\right) dx-\mathrm{NI}$, where N_a_, N_c_, NI and, $\mathrm{L}\_\mathrm{perovskite}$ are anion density, cation density, total ion density and perovskite thickness, respectively. We set up our model with materials and device parameters that are representative of values reported in the literature, particularly for MAPbBr_3_. The input parameters used in the simulation are listed in Table 1.

**Table 1:** Parameters used in DDM for PeLED.

Parameters	HTL	Perovskite	ETL	Unit
Thickness	90	150	90	nm
Bandgap	2.8^20^	2.3^21^	3.3^22^	eV
Affinity	2.2^23^	3.4^24^	4^25^	eV
Electron mobility	~ 0.01^26^	10^27^	100^22,27^	Cm^2^ V^−1^ s^−1^
Hole mobility	0.1^[calibrated]^	10^27^	25^22,27^	cm^2^V^−1^ s^−1^
Doping concentration	10^18^^27^	10^15^	10^18^^27^	cm^−3^
Conduction band effective density of states	10^18 [calibrated]^	10^17 [calibrated]^	10^18^^27^	cm^−3^
Valance band effective density of states	10^19 [calibrated]^	10^17 [calibrated]^	10^18^^27^	cm^−3^
Spontaneous emission lifetime	–	20 × 10^–9^	–	s
Auger recombination factor for electron and hole	~ 10^–28^^28^	~ 10^–28^^28^	~ 10^–28^^28^	cm^6^ s^−1^
SRH recombination lifetime	5 × 10^–6^^29^	5 × 10^–7^^29^	5 × 10^–6^^29^	s
Anion and cation density	–	0–10^19^	–	cm^−3^

## Results and discussion

**Figure 2:**
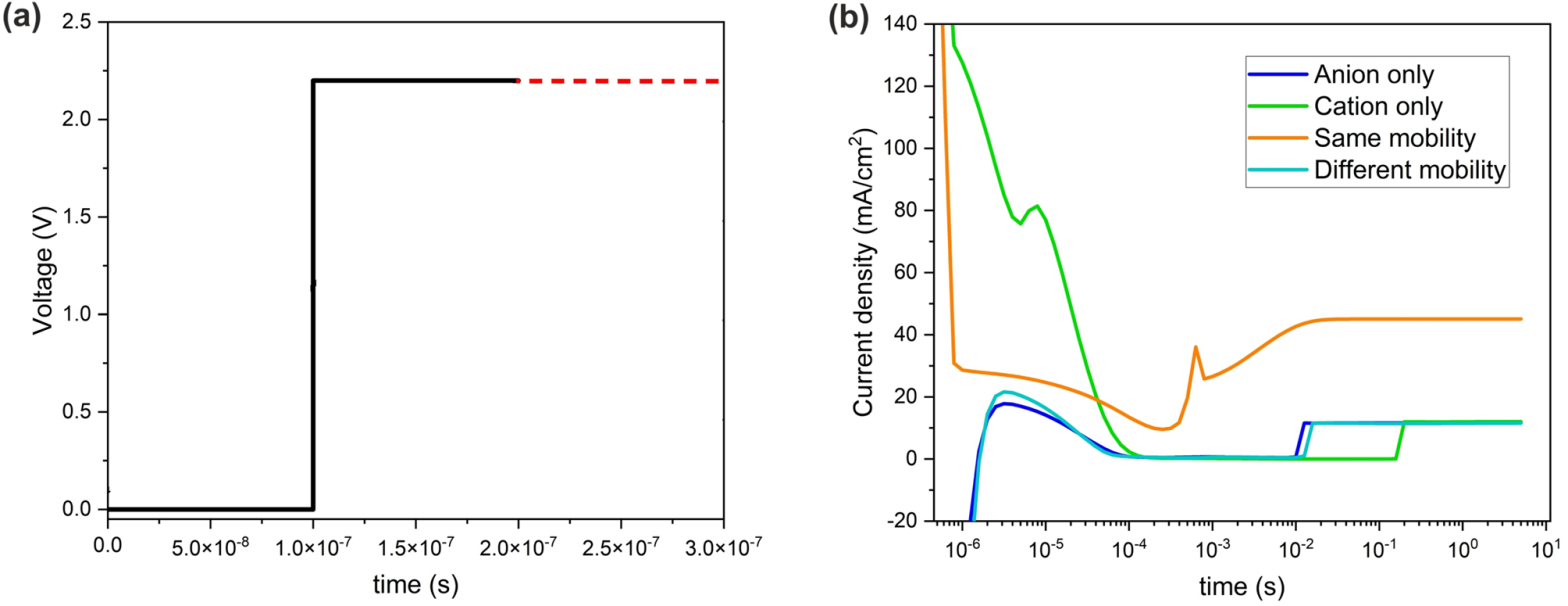
(**a**) A schematic of the step voltage, where the voltage of 2.2 V from 0.1 μs to 5 s is applied to PeLED; (**b**) The transient current of the device considering anion only, cation only, same mobility and different mobility cases at ion density of ${10}^{19} \, {{\text{cm}}}^{-3}$ and 2.2 V step voltage (threshold).

**Figure 3:**
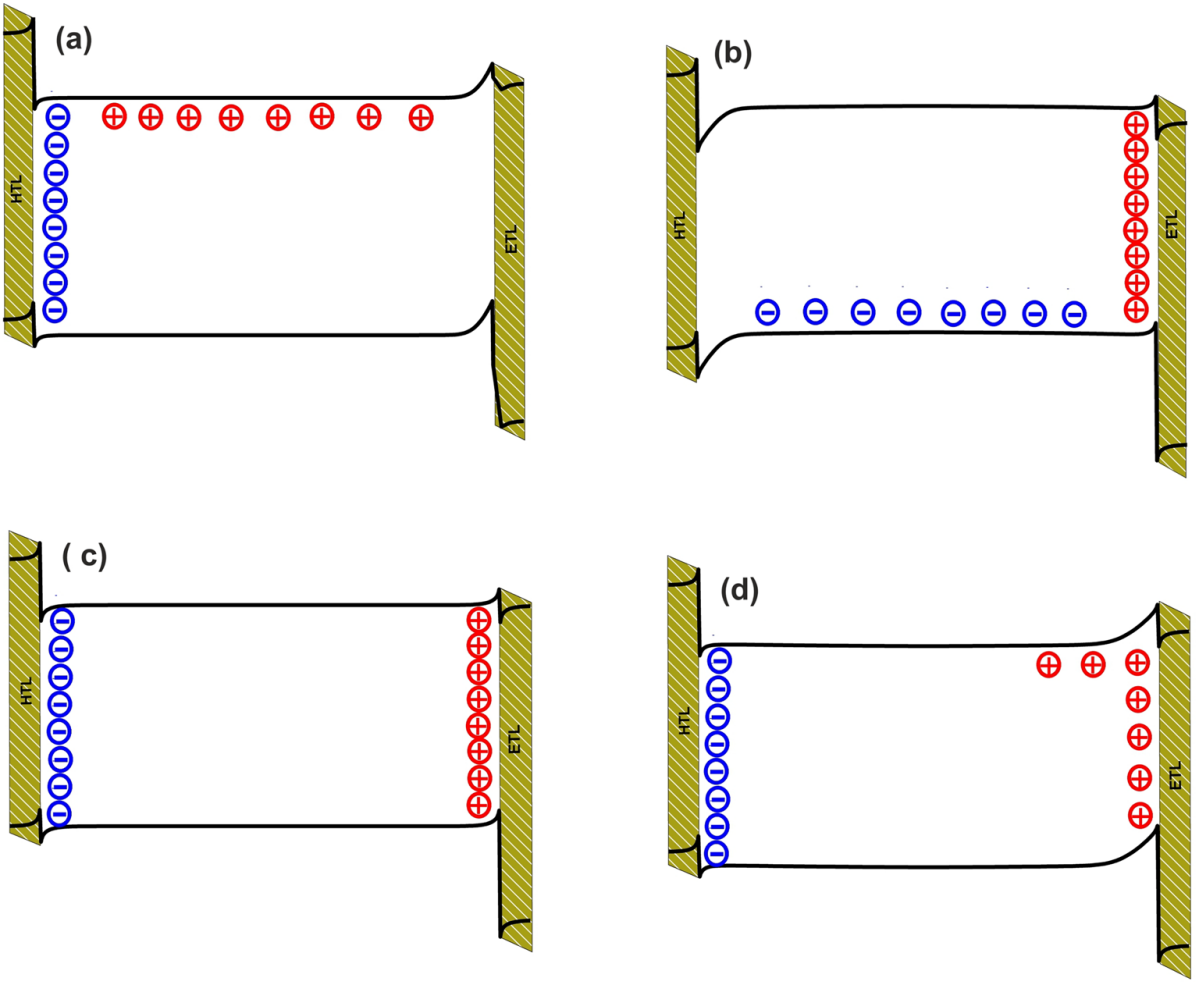
The potential surfaces and the schematic of ion distribution of the PeLED at quasi-steady-state for (**a**) anion only, (**b**) cation only, (**c**) same mobility, and (**d**) different mobility cases.

We have investigated the sub-second transient behavior of a PeLED under an applied step voltage of the device threshold voltage (2.2 V) (Fig. 2a). It has been reported that perovskite constituent ions migrate and redistribute under external bias^4,12,30^. Yet, there are ongoing discussions over the active (i.e., mobile) ion species involved in the migration process. Presuming the migration of single mobile ionic specie is the most straightforward scenario. In the case of "anion-only" merely anions (i.e., bromides) can migrate within the perovskite. These mobile ions are compensated by uniformly disturbed counterions in the background. The potential surfaces and the schematic of ion distribution of the above-described situation at quasi-steady-state (i.e., 5 s) are presented in Fig. 3a. Cation accumulation at HTL interface at the sub-second regime, hinders hole injection. As one can see from Fig. 2b, hole-depleted perovskite results in a current rise at tens of ms. “Cation-only” refers to the case where merely cations (i.e., methylammoniums) are mobile, compensated by stationary anions (Fig. 3b). In this case, anion accumulation at the ETL interface with perovskite at the sub-second regime hinders electron injection. Hence, the current rise, in this case, takes place at 100 ms. Assuming the motion of both anions and cations provides a more reliable and practical approach to investigating the impact of ion migration on the operation of the device. The potential surfaces and ion distribution of "same mobility" and "different mobility" cases are presented in Fig. 3c and d. In the former case, ${Br}^{-}$ and its vacancy (${V}_{{Br}^{-}}$) with the same mobility are introduced to the model. Therefore, anions and cations simultaneously reach the HTL and ETL interfaces with perovskite. As a result, the ion-migration-induced band bending at ETL/HTL interfaces with perovskite is symmetrical and semi-balanced charge carrier injection is expected. Hence, the current rise time reduces to a few ms. In the latter, it is assumed that ${Br}^{-}$ and ${MA}^{+}$ of different mobilities can migrate across the perovskite. The results show that the mobility difference of cations and anions (${\mu }_{c}={10}^{-12}\mathrm{\,and\,}{\mu }_{a}={10}^{-8} \, {\text{cm}}^{2}/({\text{Vs}})$, respectively^16,27^) leads to time-dependent charge carrier injection which in turn gives rise to time-transient behavior of the resultant current–time response. The low activation energy for anions migration results in immediate response to the applied electric field, hence, fast migration. Although the electric field variation induced by ion migration within the perovskite modifies the charge carrier injection properties of carrier transporting layers, there are no interaction among anions (bromide ions) and cations (methylammonium ions). Furthermore, the entrapment of charge carriers by mobile ions is not taken into account^19^.

**Figure 4:**
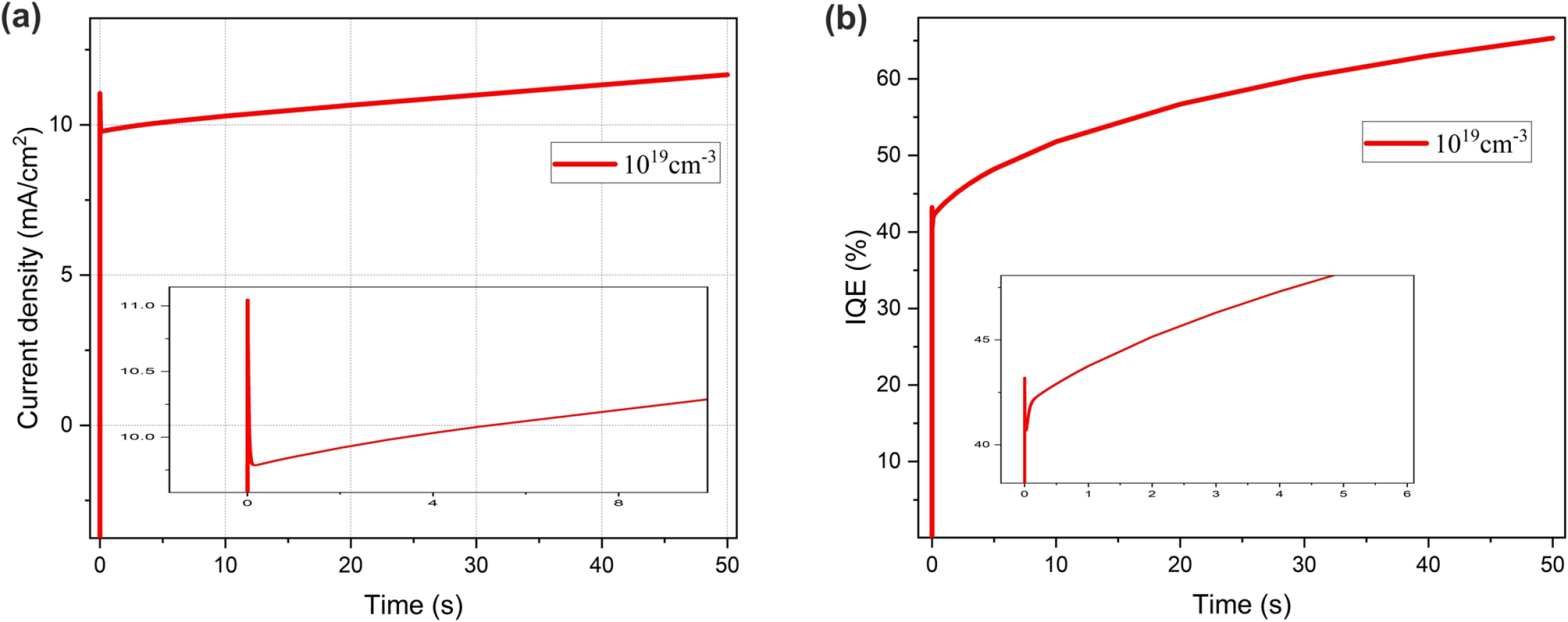
(**a**) The transient current density (Tr-J); (**b**) The transient internal quantum efficiency (Tr-IQE) of the PeLED at ion density of ${10}^{19} \, {{\text{cm}}}^{-3}$ and 2.2 V step voltage.

As a practical case, ions with different mobility have been scrutinized. To study the impact of ion migration on the device operation mechanism the transient current density (Tr-J) and transient internal quantum efficiency (Tr-IQE) of the PeLED with ion density of ${10}^{19} \, {\text{{cm}}}^{-3}$ with a step voltage of 2.2 V are presented in Fig. 4. Tr-J (Fig. 4a) shows a sharp peak during the initial time and a slow enhancement after ms, which reaches its steady-state value in a few seconds. Tr-IQE (Fig. 4b) shows a peak at 1 ms, followed by an enhancement at few seconds. Ion migration is widely accepted to be the main origin of the typical transient response of perovskite-based devices^7^.

**Figure 5:**
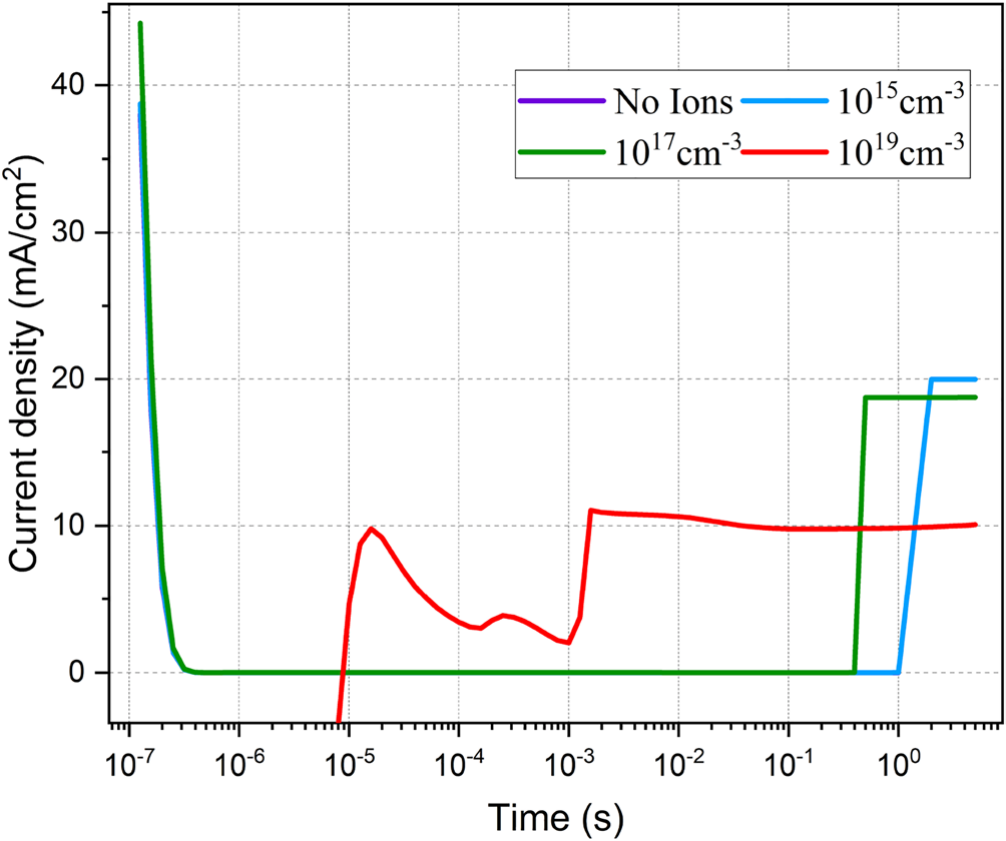
The transient current density (Tr-J) for various ion densities at 2.2 V step voltage.

To ascertain how ion migration affects the performance of PeLED, we compare the Tr-J of the devices considering various mobile ion densities on a broad dynamic range (0.1 µs–5 s) in response to the voltage step. As illustrated in Fig. 5, the 'neat' PeLED (i.e., the device without mobile ions) exhibits no transient behavior. As ion density increases (to the ${10}^{18} \, {\text{{cm}}}^{-3}$ and higher) ion migration kicks in, resulting in the current phase evolution. The sharp current rise in response to the step voltage is an indication of low surface recombination. Hence, the accumulation of anions and cations at HTL and ETL respectively, reduces the number of surface states leading to enhanced surface passivation^19^.

**Figure 6:**
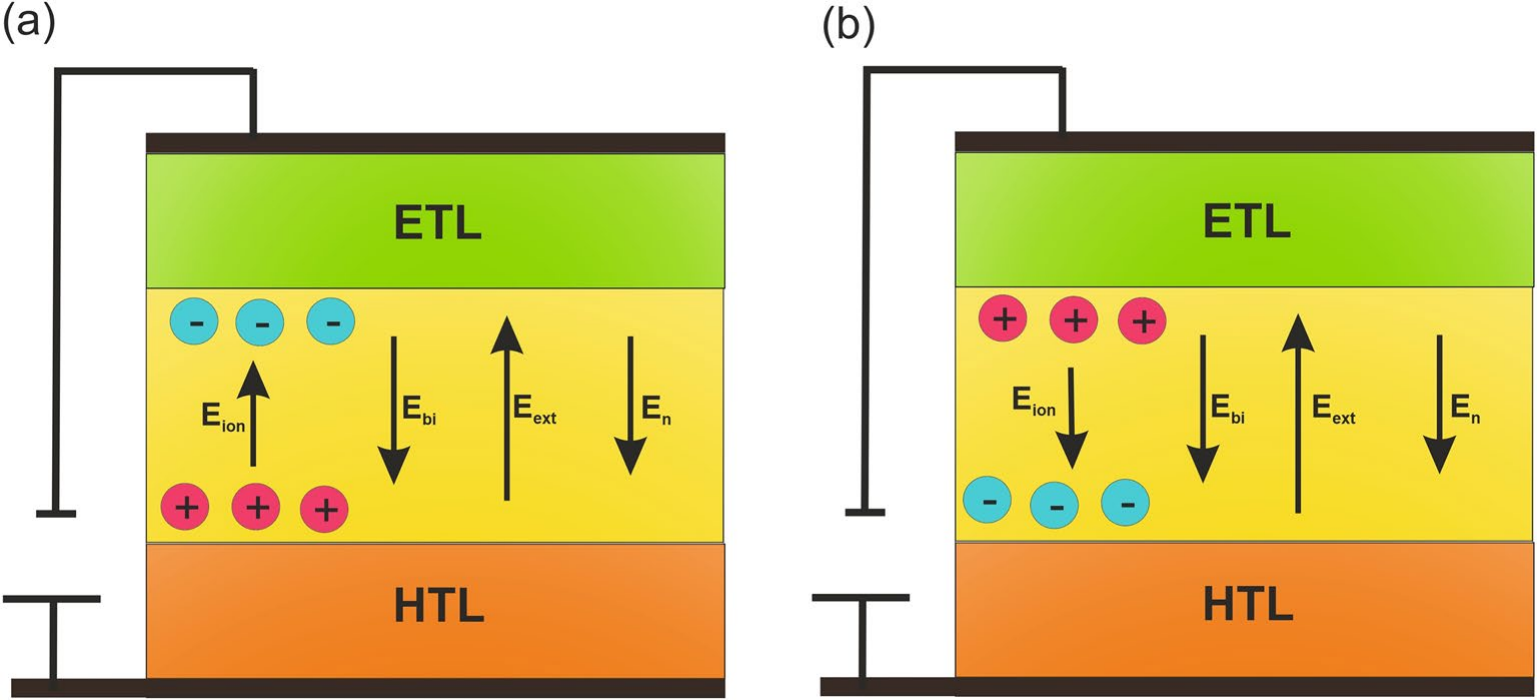
Diagram of the cations and anions behaviors under a net, built-in, external, and ion-induced- electric fields (**a**) at zero bias; (**b**) at forward bias.

Figure 6 demonstrates a diagram of the cation and anion behavior associated with various electric fields in the PeLED. The net electric field ($${E}_{n})$$ within the PeLED consists of applied voltage associated external electric field ($${E}_{ext})$$, a built-in electric field ($${E}_{bi})$$ arising from PeLED structure, and an ion redistribution electric field ($${E}_{ion})$$^4,31^. Before applying voltage, the difference in anode and cathode work functions results in a net electric field in the opposite direction of an operating PeLED^11,32,33^. This field derives anions and cations to perovskite interfaces with ETL and HTL, respectively (Fig. 6a). At short time intervals, the net electric field is $${E}_{n}={E}_{bi}-{E}_{ext}-{E}_{ion}$$. It correlates to the electric field within the perovskite being screened^32,33^. Throughout time evolution ion migration and redistribution at interfaces improves $${E}_{n}$$, which results in improved current and charge carrier recombination (i.e., $${E}_{n}={E}_{bi}-{E}_{ext}+{E}_{ion}$$) (Fig. 6b).

**Figure 7:**
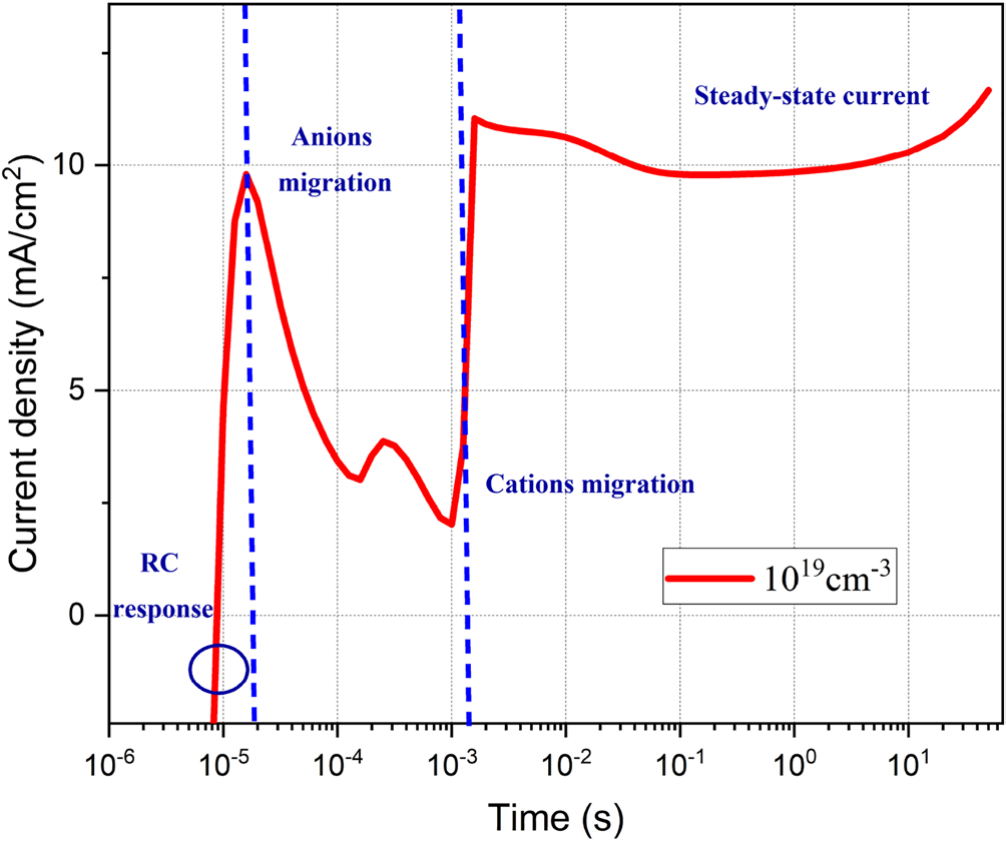
The transient current density (Tr-J) of the PeLED at ion density of ${10}^{19} \, {\text{{cm}}}^{-3}$ and 2.2 V step voltage.

In order to differentiate the influence of each ionic species migration on the transient responses of the PeLED, we have divided the J–t curve (1 µs–50 s into different phases (Fig. 7). The ion density is fixed at ${10}^{19} \, {{\text{cm}}}^{-3}$. The first phase is attributed to the initial RC response of the device equivalent circuit. Inrush current (with a significant magnitude compared to steady-state value) overshoots the device at very short time intervals (a few µs) and charges the geometrical capacitor during the power-up. The second phase comes along with the current drop as soon as the anions migrate. Since anions have lower activation energy than cations, they can instantly respond to the applied voltage and move away from the perovskite/ETL interface in a very short time. While cations accumulate at HTL/perovskite interface creating a strong electric field at the interface. This cation-induced electric field hinders hole injection^19^. Before the third phase, the current is generated by only one carrier injection. At the third phase, after ms, cations move away from the HTL/perovskite interface, and finally, ${E}_{ext}$ derives anions and cations to the perovskite interfaces with HTL and ETL, respectively. Anions at HTL and cations at ETL interfaces both lower the hole and electron injection barrier^34^. At few seconds, the PeLED reaches its steady-state operation.

**Figure 8:**
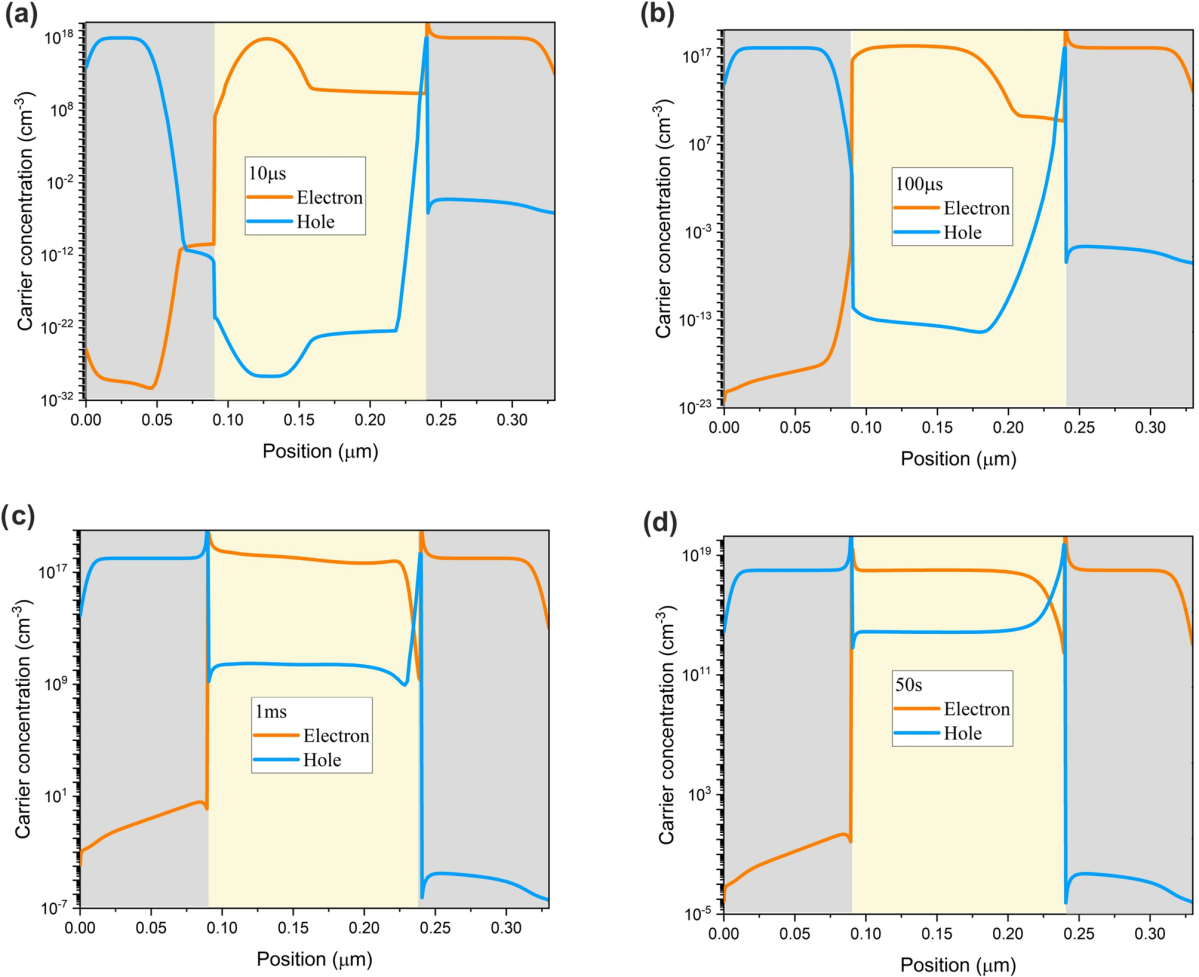
PeLEDs electron and hole concentration profiles at the (**a**) first, (**b**) second, (**c**) third phase, and (**d**) steady-state condition at ion density of ${10}^{19} \, {{\text{cm}}}^{-3}$ and 2.2 V step voltage.

Intending to illustrate the origin of the transient behavior, charge carrier concentration profiles at the first, second, and third phases and steady-state condition are plotted in Fig. 8. Once the voltage is applied, at the first phase, a significant density of electron is injected into the active layer (Fig. 8a), generating a current peak at 10 µs. In the second phase, anions migration from the perovskite/ETL interface results in the facilitation of electron injection (Fig. 8b). However, the cation-induced electric field hinders hole injection and the unbalanced charge carrier injection leads to a current drop at this phase. As cations move away from HTL/perovskite interface, the hole injection facilitates at the third phase (Fig. 8c). Hence, the current rises until it reaches the steady-state value in few seconds (Fig. 8d). Surface recombination determines the rise time of the steady-state current^19^.

**Figure 9:**
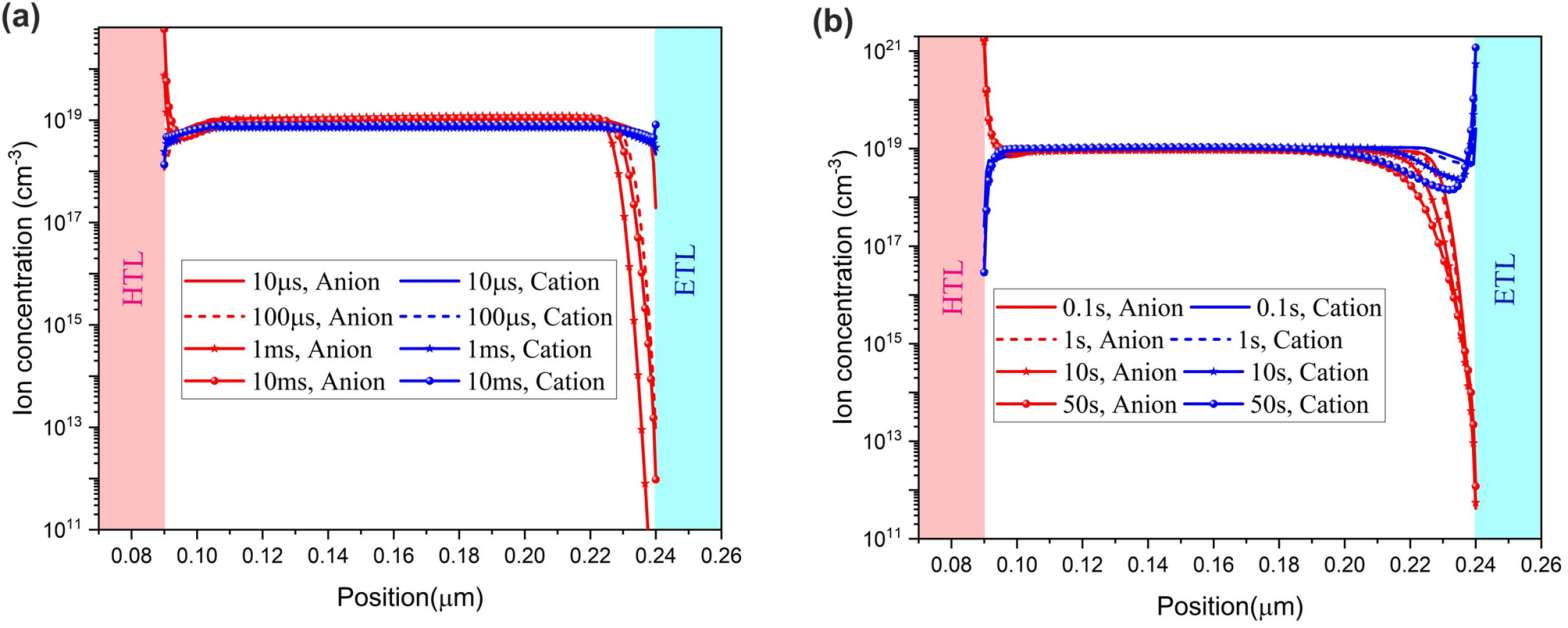
Ion migration process within the active layer of the PeLED at different time intervals (**a**) from 10 µs to 10 ms; (**b**) from 0.1 s to 50 s at ion density of ${10}^{19}\, {\text{{cm}}}^{-3}$ and 2.2 V step voltage.

To further investigate the delay between the migration time of anions and cations, ionic density profiles at different time intervals are presented in Fig. 9. Before the step voltage, ion distribution within the perovskite is nearly uniform. Even yet, ${E}_{bi}$ derives a small number of anions and cations to the perovskite ETL and HTL interfaces. At very short times (i.e., 10–100 µs, anions start to migrate from the perovskite/ETL to the perovskite/HTL interface (Fig. 9a) while the cations migration due to their low mobility is negligible. At 1–10 ms, cations start to move away from perovskite/HTL to perovskite/ETL interface) and cation migration improves ${E}_{n}$ within the perovskite. The restoring forces provided by ${E}_{n}$ and polarized perovskite derives anions to perovskite/ETL interface. At 0.1 to 50 s, ion reduction at the perovskite body and their accumulation at perovskite interfaces are discernible (Fig. 9b).

**Figure 10:**
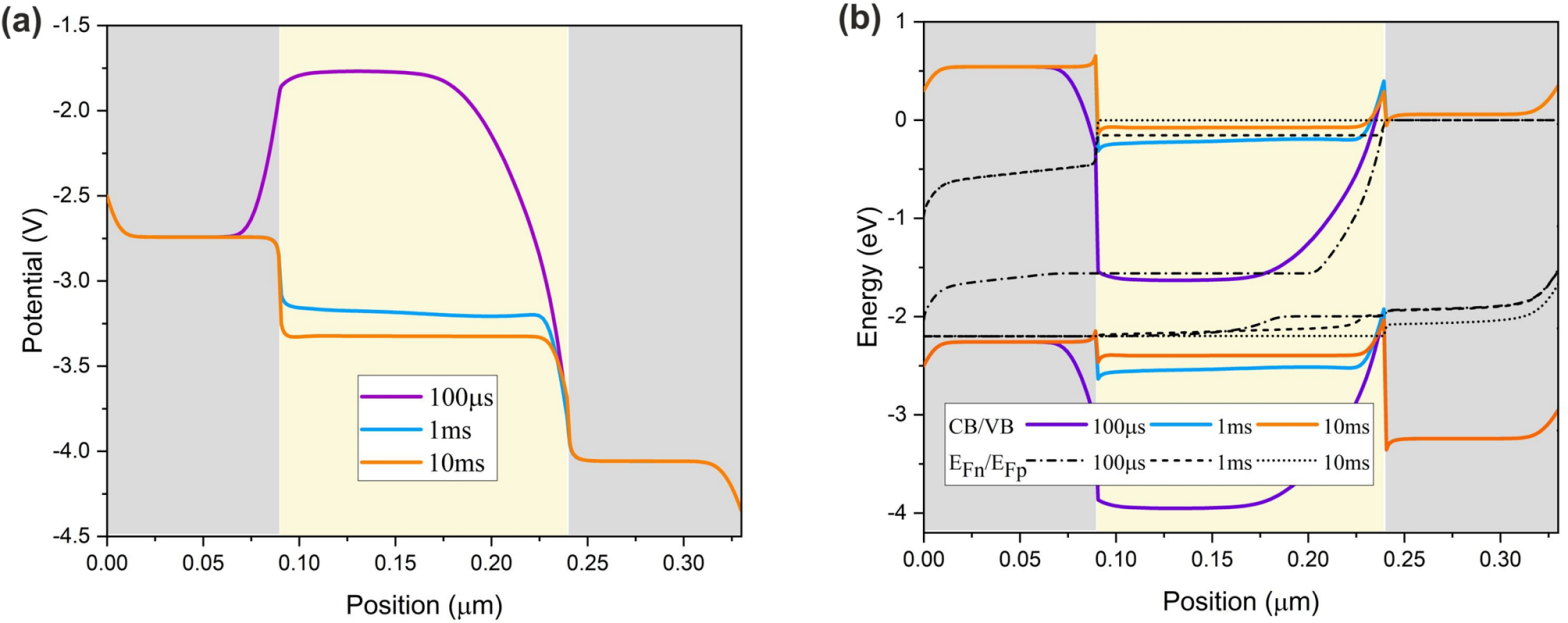
(**a**) Potential and (**b**) band alignment of the PeLED at 100 µs, 1 ms, and 10 ms at ion density of ${10}^{19} \, {{\text{cm}}}^{-3}$ and 2.2 V step voltage.

**Figure 11:**
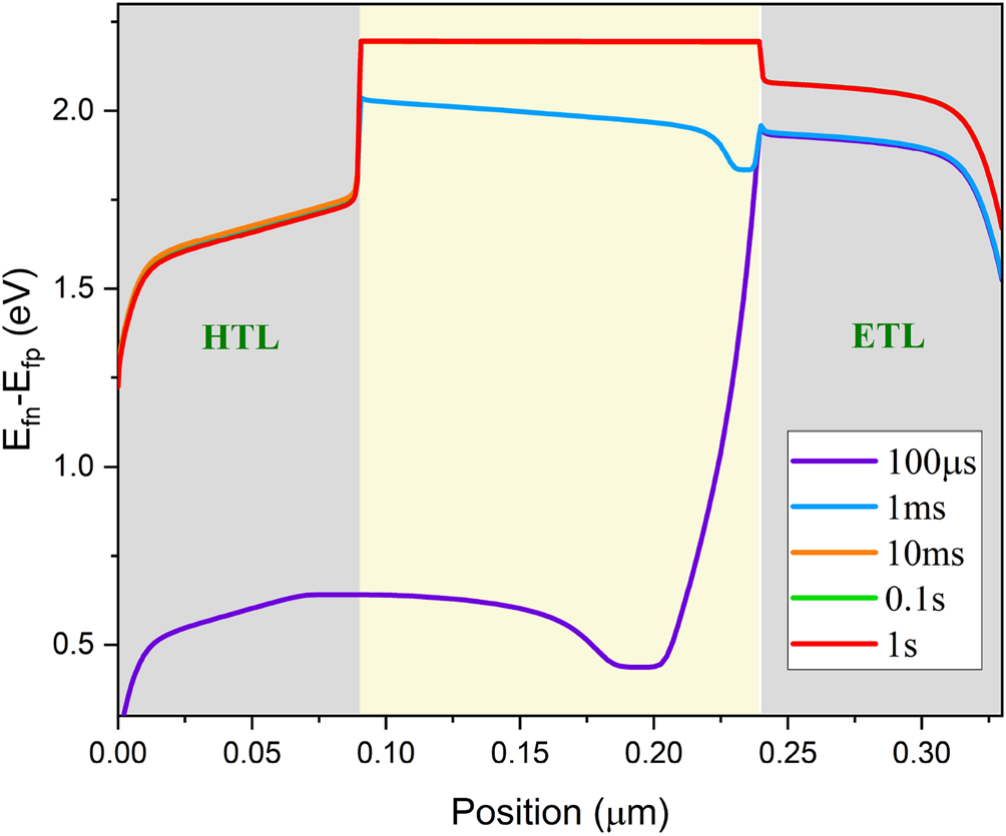
Difference of the quasi-fermi levels at different time intervals at ion density of ${10}^{19} \, {{\text{cm}}}^{-3}$ and 2.2 V step voltage.

To get a more comprehensive impression of each ionic species motion on the device operation, the potential and band alignment of the PeLED is studied. Figure 10 represents the potential and band alignment of the PeLED at 100 µs, 1 ms, and 10 ms. Anions have moved away from the perovskite/ETL interface from 100 µs to 1 ms (i.e., during the second phase). Consequently, a significant reduction in the potential barrier to electron injection occurs. On the other hand, cation accumulation at the perovskite/HTL interface generates an electric field in the opposite direction of ${E}_{ext}.$ This field gives rise to asymmetrical potential profile and band bending. Moreover, the hole injection barrier is high at 100 µs which leads to unbalanced charge carrier injection and hence, current drops. As cations depart from the interface over time, the hole injection will be facilitated during the third phase (1 ms to 10 ms) where the balanced charge carrier injection leads to the current rise. Furthermore, the law of mass action indicates that $np={n}_{i}^{2}(\mathrm{exp}\left(\frac{\Delta \eta }{V}\right)-1)$, where $\Delta \eta$ and V are the separation of the quasi-fermi levels and applied voltage^35^. $n, p,$ and ${n}_{i}$ are electron, hole, and intrinsic carrier concentration, respectively. For longer time intervals, $\Delta \eta$ increases leading to the higher injected carrier density and current rise. Achieving balanced charge carrier injection puts an end to quasi-fermi level splitting (Fig. 11) resulting in the steady-state operation of PeLED at the constant applied voltage.

**Figure 12:**
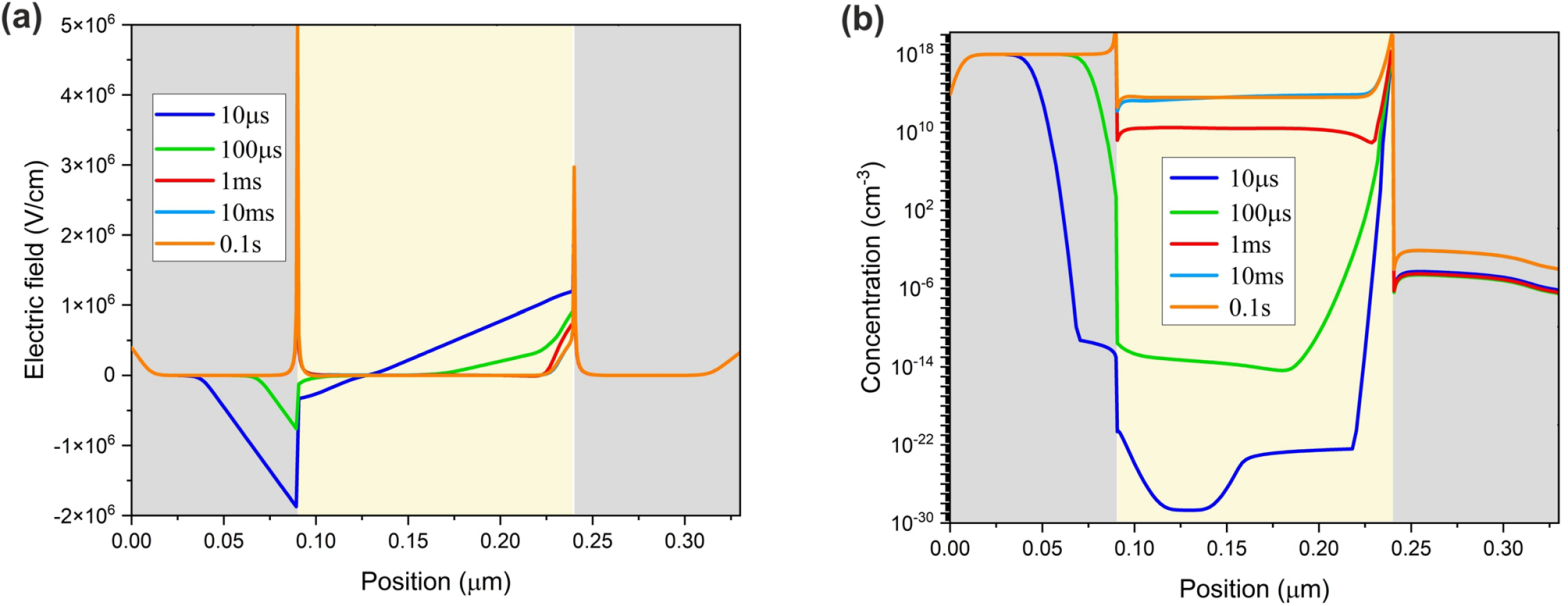
(**a**) The electric field at different time intervals; (**b**) The hole concentration at equivalent time intervals within the perovskite at ion density of ${10}^{19} \, {\text{{cm}}}^{-3}$ and 2.2 V step voltage.

To further verify the suggested ion-migration-induced delay in the hole injection mechanism, we investigate the resultant electric field. At short time intervals, such as 10 µs, cation accumulation at HTL/perovskite interface screens the electric field. As anions reach this interface under ${E}_{ext}$, the electric field modifies. However, this modification is not substantial. Entering the third phase (i.e., 1 ms), cations move away from the interface, resulting in significant electric field enhancement (Fig. 12a). Hole concentration at equivalent time intervals is presented in Fig. 12b. A symmetrical charge carrier injection profile results from efficient hole injection at the third phase.

**Figure 13:**
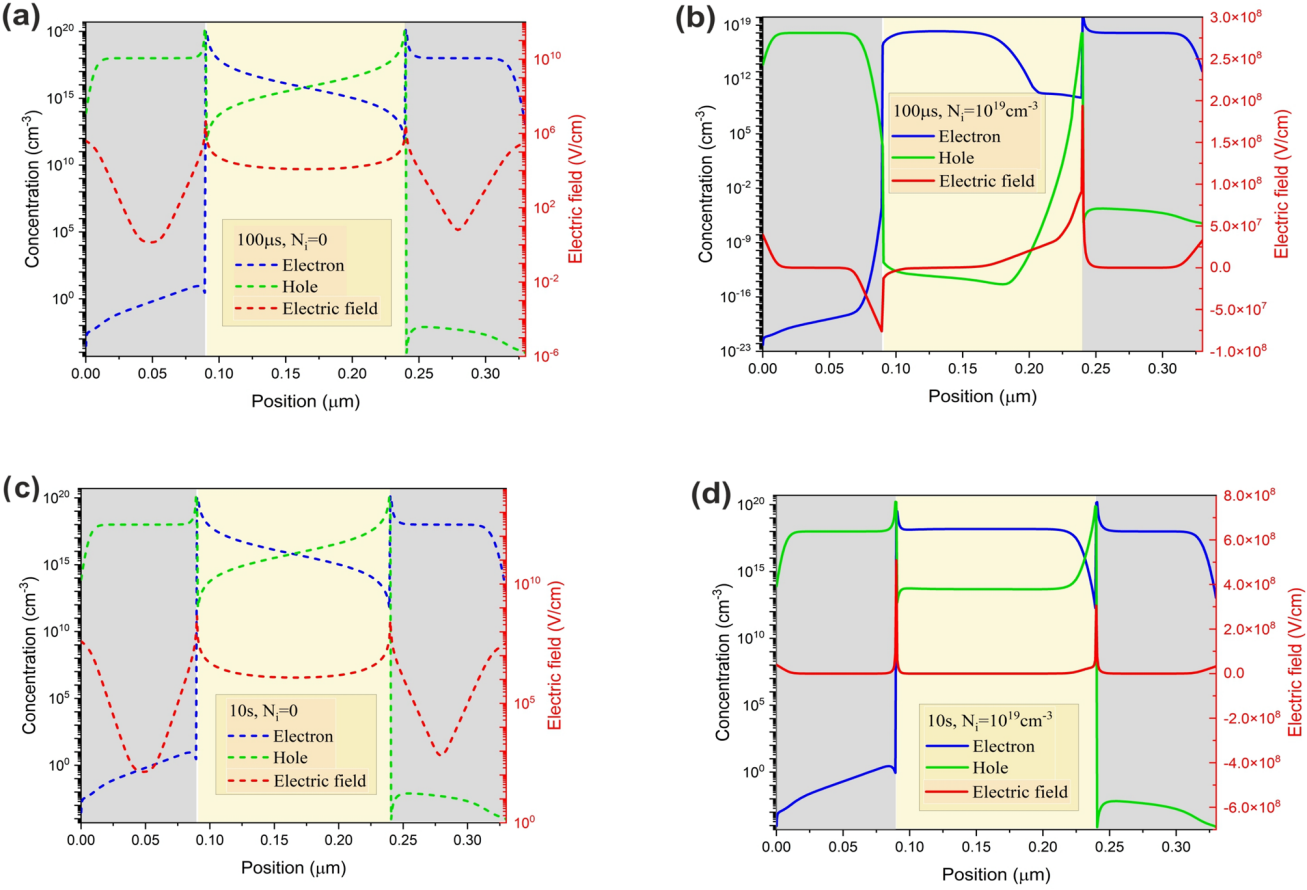
The electric field and consequent carrier concentration at (**a**) 100 µs for the neat device; (**b**) 100 µs for the device at ion density of ${10}^{19} \, {\text{{cm}}}^{-3}$; (**c**) 10 s for the neat device; (**d**) 10 s for the device at ion density of ${10}^{19}$.

**Figure 14:**
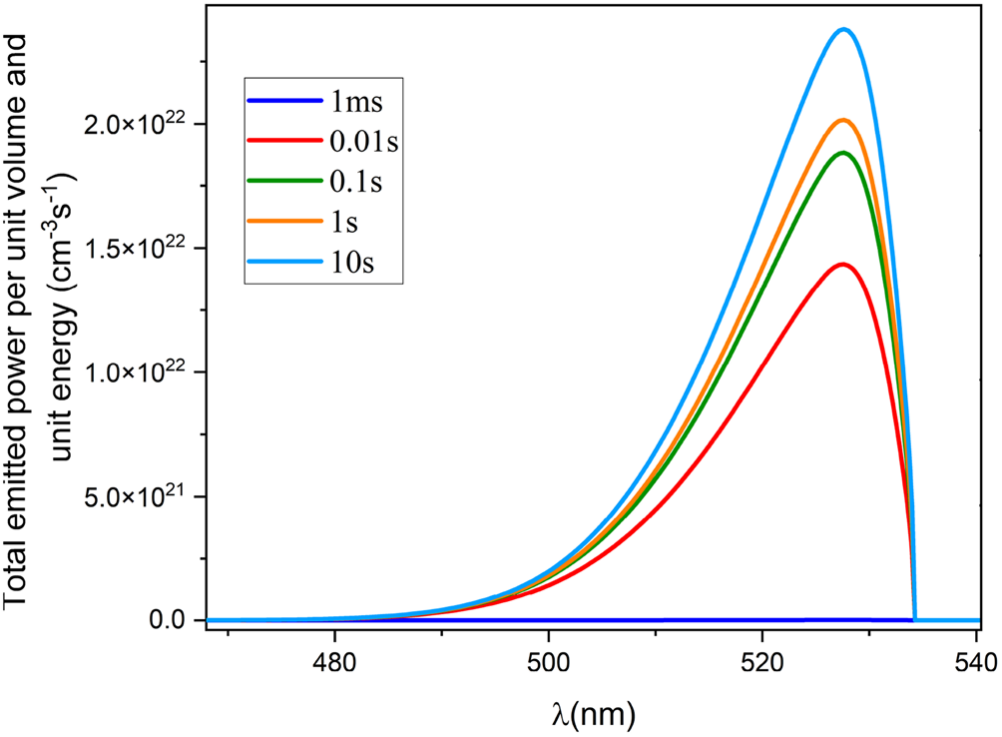
Total emitted power per unit volume unit energy at different time for the device with ion density of ${10}^{19} \, {{\text{cm}}}^{-3}$ under 2.2 V step voltage*.*

Figure 13 presents the electric field and consequent carrier concentration at 100 µs and 10 s, in the neat device and the device with ion. Unbalanced charge carrier injection from ETL and HTL is observable. The electron mobility for ZnO is 100 cm^2^/(V s), which is three orders of magnitude higher than the hole mobility for PEDOT:PSS (0.1 cm^2^/(V s)). The mobility mismatch of the transport layers leads to timely^36^ and unbalanced charge carrier injection^37,38^. Furthermore, in the case of ion migration, (Fig. 13b,d) dynamic ion redistribution within the perovskite changes the net electric field of the device. Moreover, Cation and anion mobility mismatch leads to asymmetrical electric field profile at short time intervals. The unbalanced carrier injection results in capacitance behavior and hysteresis in PeLEDs^39^. As the spatial distribution of the electric field takes on a symmetrical form, the device achieves steady-state operation and semi-balanced charge carrier injection. The net electric field determines the total emitted power from PeLED. Immediately, after the step voltage application, the intensity of the emitted power is weak. Ion redistribution within the perovskite improves ${E}_{n},$ which leads to better charge carrier injection, higher carrier density favoring radiative recombination, and hence more emitted power (Fig. 14).

**Figure 15:**
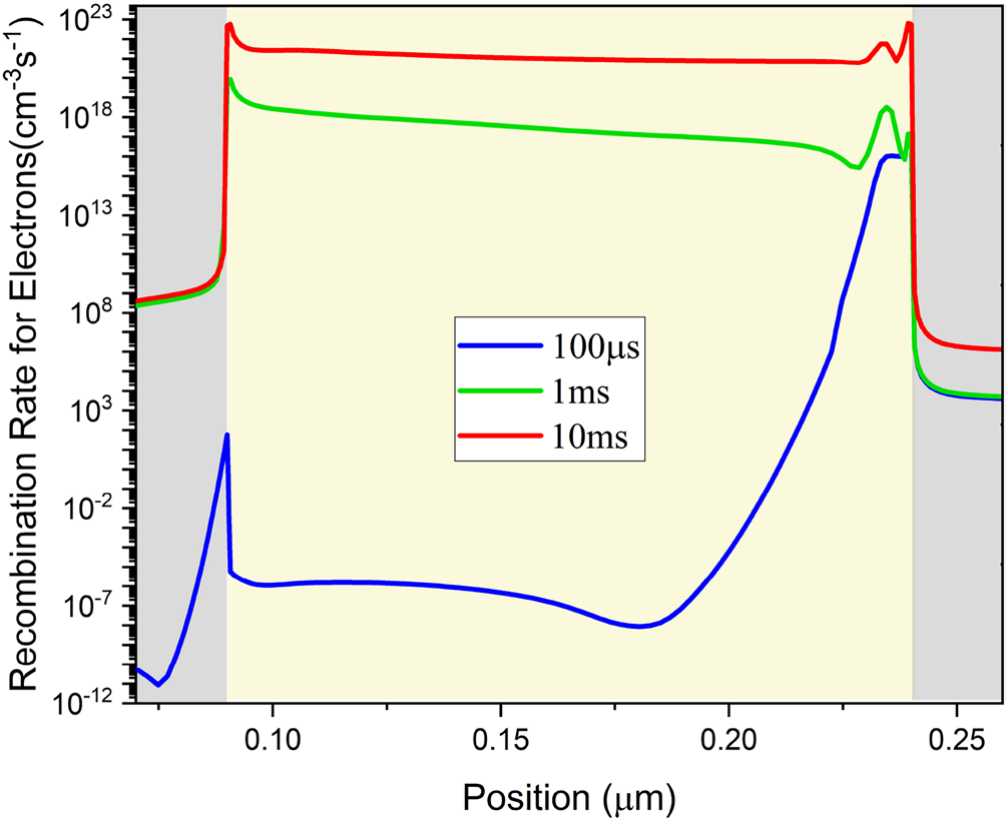
Recombination rate for electrons within the perovskite at 100 μs, 1 ms and 10 ms at ion density of ${10}^{19} \, {\text{{cm}}}^{-3}$ and 2.2 V step voltage.

The electron recombination rate within the perovskite at 100 µs, 1 ms, and 10 ms has been shown in Fig. 15. Within short time intervals, recombination is highly asymmetric and strong at the Perovskite/ETL interface. At longer times, its profile becomes uniform and stays unvarying within the perovskite thickness.

**Figure 16:**
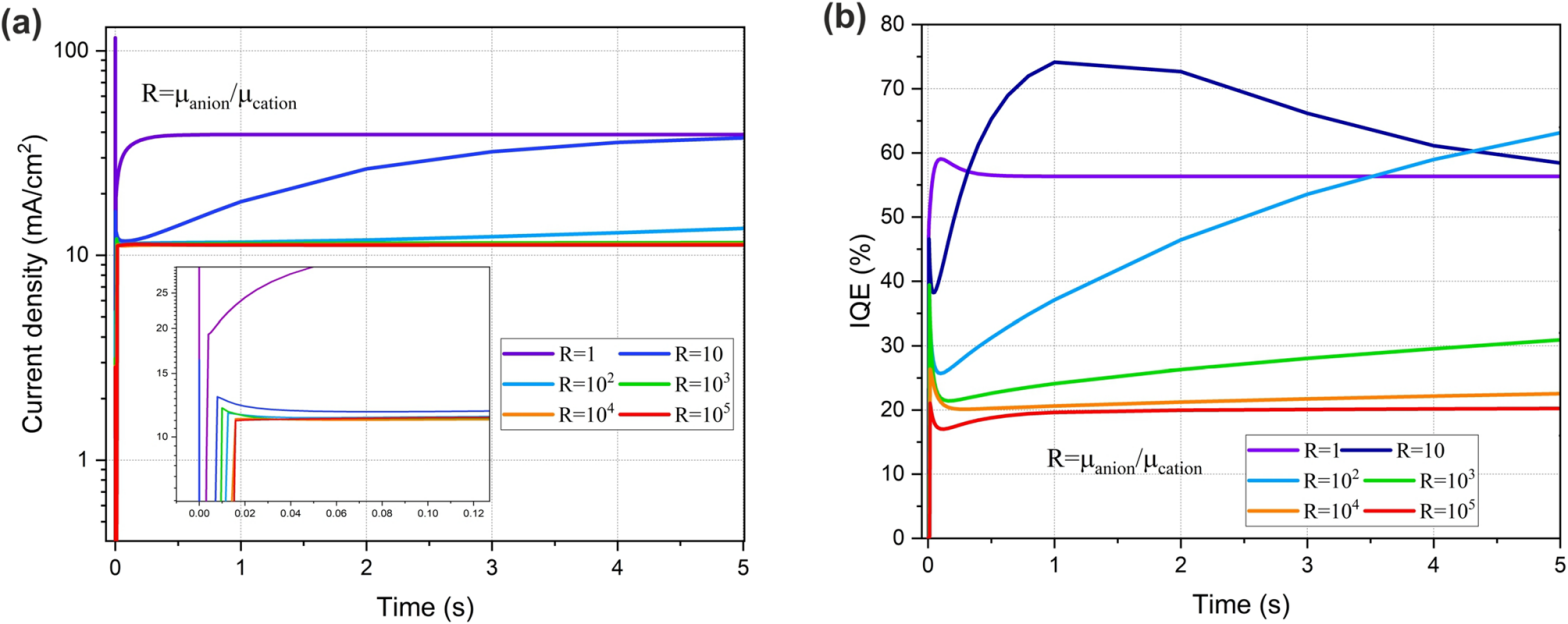
The influence of the ratio of ion mobility, $R=\frac{{\mu }_{anion}}{{\mu }_{cation}}$ on the time-transient behavior of PeLED at ion density of ${10}^{19} \, {\text{{cm}}}^{-3}$ and 2.2 V step voltage, (**a**) Current density-time response and, (**b**) IQE-time of the PeLED.

Ion mobility and diffusion play an important role in the transient response of the PeLED. Our findings indicate that the mobility difference between cations and anions is the main root of the transient response of the PeLED. Diffusion coefficients for cation and anion (${D}_{ion}$) at T = 297 K are calculated from the Einstein relation as depicted in Eq. (6):

6 $${D}_{ion}={\mu }_{ion}\left(\frac{{k}_{B}T}{e}\right)$$

where ${\mu }_{ion}, {k}_{B},T$ and e are ion mobility, Boltzmann constant, temperature and elementary charge, respectively. We define $R={\mu }_{anion}/{\mu }_{cation}$. Tr-J response of the PeLED with ion density of ${10}^{19} \, {\text{{cm}}}^{-3}$ and 2.2 V step voltage for fixed anion mobility $of {10}^{-8} \, {\text{ {cm}}}^{2}/({\text{Vs}})$ and various cation mobility is presented in Fig. 16. For slow cations (R > 10^3^), electron injects inconvincibly over a longer period of time. So, it takes a longer time for the current to rise and the steady-state current reduces significantly. Moreover, the Tr-IQE (Fig. 16b) reveals that the slower cations migration which is responsible for the hole-depleted perovskite, results in lower radiative recombination rate, hence lower IQE of the PeLED. It has been proposed that the slow-migrating cations are the origin of hysteresis^14,16^. Our findings suggest that the time delay between the electron and hole injection leads to hysteresis in the PeLEDs. In the case of applying a low scan rate, both electrons and holes can follow the electric field changes, reducing the injection time delay, and leading to minimal hysteresis in the perovskite-based devices.

## Conclusions

In summary, a comprehensive study on the role of ion migration on the time-transient performance of a PeLED has been carried out. The results show that anions and cations migrate in different time scales (microseconds to a few seconds) due to their mobility difference, which brings about time-transient electric field redistribution, dynamic charge carrier injection, and slow response of current. Timely migration of cations and anions leads to a time delay between electron and hole injection. Unbalanced charge carrier injection might be the main root of anomalous hysteresis for the perovskite-based devices. Scan rate, as an important parameter, controls the charge carrier injection time. Hence, the application of an appropriate scan rate might significantly reduce the hysteresis. Since the steady-state performance of the PeLED is strongly affected by sub-second ion migration-induced phenomena, time-transient investigation of the underlying process in short times is strongly advised. This research offers new insights into the origins of reduced hysteresis in PeLEDs, which can be achieved by cation migration suppression.

## Data Availability

The data that support the findings of this study are available from the corresponding author, [E.Y.], upon reasonable request.
